# Negative impact of proteinuria on circulating myeloid dendritic cells

**DOI:** 10.1007/s10157-019-01724-7

**Published:** 2019-03-16

**Authors:** Masato Ikeda, Risa Terashima, Taku Yamada, Masahiro Suyama, Shinya Yokote, Masatsugu Nakao, Izumi Yamamoto, Keita Hirano, Hideo Okonogi, Hiroyasu Yamamoto, Takashi Yokoo

**Affiliations:** 1 Division of Nephrology and Hypertension, Department of Internal Medicine, Katsushika Medical Center, Jikei University School of Medicine, 6-41-2 Katsushika-ku, Tokyo, 125-8506 Japan; 2 Division of Nephrology and Hypertension, Department of Internal Medicine, Jikei University School of Medicine, Tokyo, Japan

**Keywords:** Dendritic cells, Proteinuria, eGFR, Gender, C-reactive protein

## Abstract

**Background:**

A decrease in absolute numbers (abs.) of circulating dendritic cells (DCs) and recruitment into target organs has been reported, but whether the level of proteinuria associates with circulating DC abs. has not been clarified.

**Methods:**

We conducted a cross-sectional study of 210 patients with kidney disease aged 21–96 years who were admitted to our hospital for kidney biopsy in 2007–2010. For accuracy, the level of proteinuria was thoroughly measured by 24-h urine collection from patients in their admitted condition. The abs. of total DCs (tDCs), myeloid DCs (mDCs) and plasmacytoid DCs (pDCs) was measured by three-color fluorescence-activated cell sorting (FACS). Patients were divided into four groups based upon the quartile of each DC abs. and one-way ANOVA, and multivariable-adjusted regression analyses were performed.

**Results:**

Quantile analysis showed that the level of daily proteinuria decreased with increasing blood mDC abs., with mean proteinuria levels (g/day) of 2.45, 1.68, 1.68, 1.10 for those in mDC abs. quartiles ≤ 445, < 686, < 907, ≥ 907 cells/10^2^ µL (*p* = 0.0277), respectively. Multivariate-adjusted regression analysis revealed that the mDC abs. was negatively associated with proteinuria (95% CI − 57.0 to − 8.5) and positively associated with male gender (95% CI 66.2–250.5). Independent associations were also shown between pDCs abs. and estimated glomerular filtration rate (eGFR) (95% CI 0.14–2.67) and C-reactive protein (95% CI − 49.4 to − 9.9) and between tDCs abs. and male gender (95% CI 54.5–253.6) and C-reactive protein (95% CI − 80.5 to − 13.4).

**Conclusion:**

We first reported that circulating mDC abs. has a negative association with the level of proteinuria.

## Introduction

DCs are the major antigen-presenting cells and are distributed throughout the body including kidney [1]. The subsets of circulating DCs include precursor mDCs, or conventional DCs that initiate T-cell immunity and antibody production, and pDCs, which have an important role in antiviral immunity and immune tolerance.

Several diseases influence circulating DCs, and decreases in circulating DCs and recruitment into target organs have been reported in autoimmune diseases [2–4], infections [5], coronary artery disease [6], central nervous system (CNS) disturbances [7, 8] and atherosclerosis, where DCs have been identified in atherosclerotic plaques [9].

In kidney mDCs and pDCs, both subsets usually locate in the tubulointerstitium often with a high frequency [10] and increase in number as the severity of kidney disease increases [11].

A reduction in the number of circulating pDCs has been reported in patients with chronic kidney disease (CKD) compared with that in healthy controls. Several reports have shown that the reduction in pDC numbers was more striking than that in mDCs [12–14]. However, the reduction in the number of circulating mDCs is controversial. Lim et al. [13] reported that circulating mDCs abs. in dialysis patients was comparable to that in healthy controls. Hesselink et al. [12] also reported that the estimated glomerular filtration rate (eGFR) was correlated with pDC numbers, but not mDC numbers, and these levels were comparable to those of volunteer patients with CKD not receiving dialysis.

Though the level of proteinuria is a sensitive marker for kidney disease, the association between the circulating DCs abs. and the level of proteinuria has not been clarified to date. If the circulating DC abs. decreases with the level of proteinuria, the measurement of blood DC abs. may be a biomarker for renal disease activity.

Considering all the evidence, we evaluated the hypothesis that the circulating DC abs. could be altered by the level of proteinuria, and we found that mDCs abs. was significantly associated with the level of proteinuria negatively, independent of eGFR. We also found that the number of pDCs, but not the number of mDCs, was associated with eGFR by multivariate regression analysis adjusted for proteinuria, C-reactive protein, and gender.

## Methods

### Study design

This study was a cross-sectional, observational, single-center study and comprised 243 Japanese patients who received kidney biopsy between December 1, 2007, and June 31, 2010. Clinical information and hematological data were collected immediately before the kidney biopsy. This study was performed in accordance with the Declaration of Helsinki. The Ethics Committee for Clinical Research of Jikei University School of Medicine approved this study [permission no. 29-032 (8648)].

**Fig. 1:**
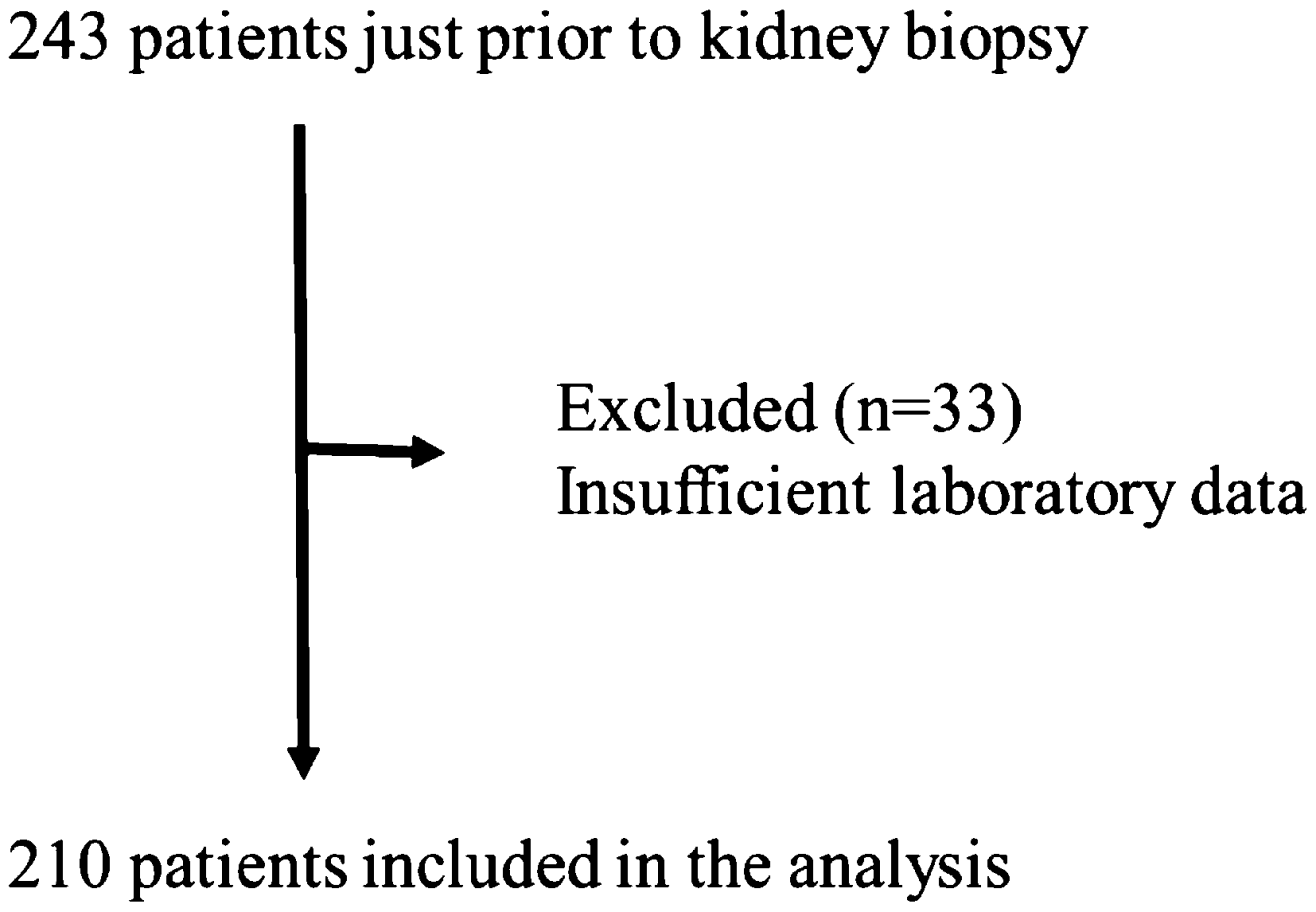
Flow chart of recruitment of study participants. Patients were excluded from the analysis due to insufficient laboratory data (33 patients). Of the 243 patients evaluated, 210 Japanese patients satisfied the inclusion criteria and were included in the analysis

To explore the association between the abs. of each subset of DCs and clinical data, the following inclusion criteria were selected: (1) adult patients with biopsy-proven kidney disease and (2) patient records with complete data for the following factors: age, gender, body mass index (BMI), glucocorticoid use, level of proteinuria in collected daily urine, and fasting blood samples. As a result, patients were excluded from the analysis due to insufficient laboratory data (33 patients). Therefore, of the 243 patients evaluated, 210 Japanese patients satisfied the inclusion criteria and were included in the analysis (Fig. 1).

eGFR was calculated using Japanese equation [15]: eGFR (mL/min/1.73 m^2^) = 194 × Cr^−1.094^ × age^−0.287^ (× 0.739 for women).

The primary variable was the abs. of mDCs, pDCs, and total DCs. Predictor variables were the levels of daily proteinuria, eGFR, C-reactive protein, and the other clinical parameters.

## Flow cytometric identification of DCs

Peripheral blood samples were collected in the morning during admission for kidney biopsy, and flow cytometric analysis and peripheral blood DC quantification were performed within 3 h after blood sampling. DCs were identified in heparinized whole-blood samples (Greiner Bio-One, Frickenhausen, Germany) using a combination of fluorochrome-conjugated monoclonal antibodies to different membrane markers. Reagents were obtained from BD Biosciences (Erembodegem, Belgium): 20 µL lineage cocktail 1-fluorescein isothiocyanate (Lin1-FITC), containing a mixture of anti-cluster of differentiation (CD) 3, CD14, CD16, CD19, CD20, and CD56 antibodies; 5 µL anti-CD123-PE; 5 µL anti-CD11c-APC; and 10 µL anti-human leukocyte antigen (HLA)-DR were incubated with 100 µL whole blood for 15 min at room temperature in the dark. Cells were fixed and lysed for 20 min at room temperature using FACS lysing solution (BD Bioscience). Cells were centrifuged at 200×*g* for 10 min and resuspended in 0.5 mL phosphate-buffered saline containing 0.1% NaN_3_.

**Fig. 2:**
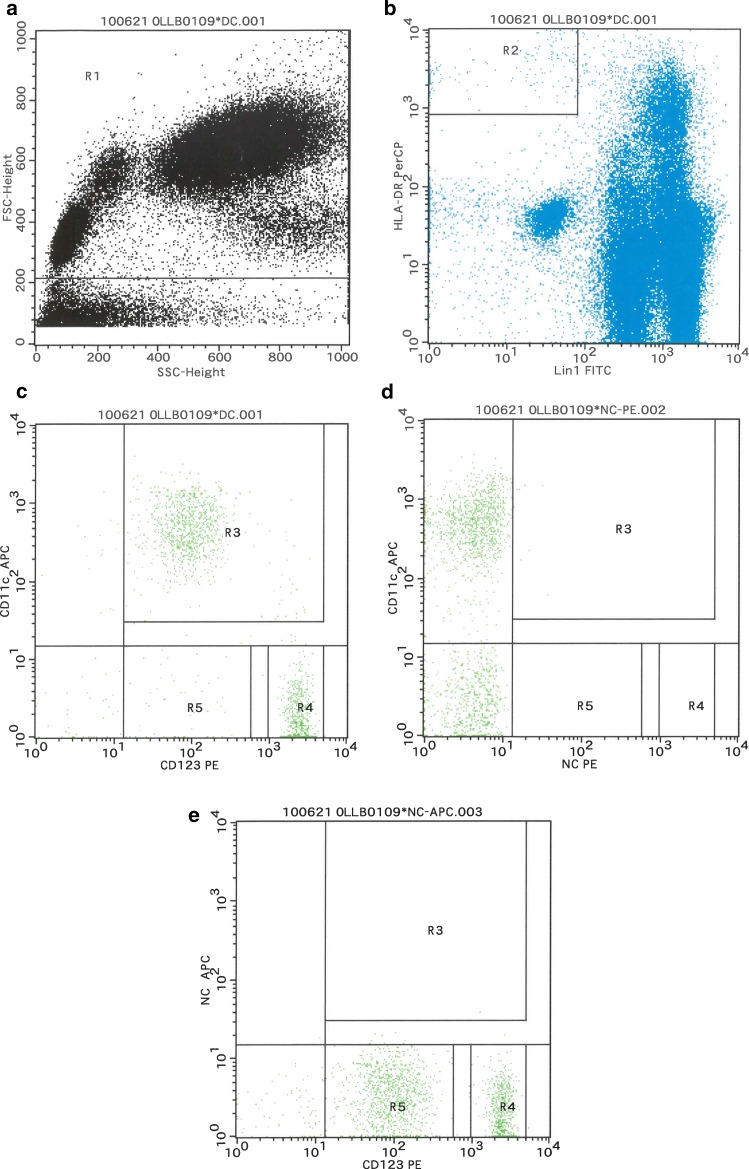
Flow cytometric identification of DCs. Representative dot plots showing the gating strategy to identify mDCs and pDCs by flow cytometry. Whole peripheral leukocytes were selected from live cells based on SSC-height and FSC-height characteristics (**a**, gate R1). Blood DCs were then selected within R1-positive cells as negative for lineage markers (lin-1) (CD3, CD14, CD16, CD19, CD20, and CD56) and positive for HLA-DR. (**b**, gate R2). Next, according to the expression of CD123 and CD11c, blood DCs were identified as mDCs (**c**, HLA-DR+/CD11c+/CD123 dim^+^, Gate R3) or pDCs (**c**, HLA-DR+/CD11c-/CD123 high^+^, Gate R4). Dot plots for the negative control (NC) of either CD 123 (**d**, NC PE) or CD11c (**e**, NC APC) are also shown

Data acquisition was performed on a three-color FACSCalibur (BD Bioscience) using a well-defined gating strategy in Cell Quest Pro software (Beckton Dickinson, Heidelberg, Germany). At least 5 × 10^5^ cells were measured to obtain a minimum of 1000 HLA-DR-positive DCs. Representative dot plots of the gating strategy for identifying mDCs and pDCs by three-color FACS are shown in Fig. 2. Whole peripheral leukocytes were selected from live cells based on SSC-height and FSC-height characteristics (Fig. 2a, gate R1). Blood DCs were then selected within R1-positive cells as negative for lineage markers (lin-1) (CD3, CD14, CD16, CD19, CD20 and CD56) and positive for HLA-DR (Fig. 2b, gate R2). Next, according to the expression of CD123 and CD11c, blood DCs were identified as mDCs (Fig. 2c, HLA-DR+/CD11c+/CD123 dim^+^, Gate R3) or pDCs (Fig. 2c, HLA-DR+/CD11c-/CD123 high^+^, Gate R4) [8, 16, 17]. Dot plots for the negative control of either CD 123 (Fig. 2d) or CD11c (Fig. 2e) are also shown.

## Statistics

Statistical analyses were performed using JMP 9.0 (SAS Institute Inc., Cary, NC, USA). Data are expressed as the mean ± standard deviation or numbers (percentage) of patients. Comparisons across the various groups were performed using the Pearson Chi square test for categorical data, Dunnett test for continuous data and ANOVA for quartile analysis. All tests were two-tailed, and a *p* value of < 0.05 was considered significant. Factors associated with DC abs. on univariate analysis were subsequently included in a multivariate model. Multivariate regression analyses were performed to identify the covariates associated with each DC abs. *T* values and 95% confidence intervals (CIs) were determined using univariate and multivariate logistic regression models for the factors that were significantly associated with each DC abs.

## Results

### Histopathological diagnoses and patient characteristics

Histopathological diagnoses of these patients including IgA nephropathy (*n* = 67, 31.9%), benign/malignant nephrosclerosis (*n* = 31, 14.8%), diabetic nephropathy (*n* = 26, 12.4%), membranous nephropathy (*n* = 15, 7.1%), minor glomerular abnormalities (*n* = 14, 6.7%), crescentic glomerulonephritis (*n* = 11, 5.2%), focal segmental glomerulosclerosis (*n* = 11, 5.2%), minimal change disease (MCD) (*n* = 7, 3.3%), lupus nephritis (*n* = 5, 2.4%), IgA vasculitis (*n* = 4, 1.9%), obesity-related nephropathy (*n* = 4, 1.9%) and others (*n* = 15, 7.1%) (Table 1). The other patient characteristics and clinical data are shown in Table 2.

**Table 1:** Histopathological diagnoses of participants

	Number of patients (%)
IgAN	67 (31.9)
Benign/malignant nephrosclerosis	31 (14.8)
Diabetic nephropathy	26 (12.4)
MN	15 (7.1)
Minor	14 (6.7)
FSGS	11 (5.2)
CreGN	11 (5.2)
MCD	7 (3.3)
Lupus nephritis	5 (2.4)
IgA vasculitis	4 (1.9)
Obesity-related nephropathy	4 (1.9)
Others	15 (7.1)
Total	210 (100)

Number and percentage of histopathological diagnoses including immunoglobulin A nephropathy (IgAN) (*n* = 67, 31.9%), benign/malignant nephrosclerosis (*n* = 31, 14.8%), diabetic nephropathy (*n* = 26, 12.4%), membranous nephropathy (MN) (*n* = 15, 7.1%), minor glomerular abnormalities (minor) (*n* = 14, 6.7%), crescentic glomerulonephritis (CreGN) (*n* = 11, 5.2%), focal segmental glomerulosclerosis (FSGS) (*n* = 11, 5.2%), minimal change disease (MCD) (*n* = 7, 3.3%), lupus nephritis (*n* = 5, 2.4%), IgA vasculitis (*n* = 4, 1.9%), obesity-related nephropathy (*n* = 4, 1.9%) and others (*n* = 15, 7.1%)

**Table 2:** Patients characteristics and clinical data

	Overall (*n* = 210)
Age (years)	57.6 ± 16.2 (21–96)
Male, *n* (%)	121 (57.6)
Diabetes mellitus nephropathy, *n* (%)	26 (12.4)
BMI (kg/m^2^)	23.5 ± 3.8 (14.7–35.2)
Glucocorticoids user, *n* (%)	24 (11.4)
Serum albumin (g/dL)	3.6 ± 0.7 (1.5–4.7)
Serum urea nitrogen (mg/dL)	24.9 ± 17.4 (6–109)
Serum creatinine (mg/dL)	1.72 ± 1.39 (0.48–8.9)
eGFR (mL/min/1.73 m^2^)	47.1 ± 28.0 (4.5–133.2)
Serum sodium (mEq/L)	141.5 ± 2.5 (133–148)
Serum potassium (mEq/L)	4.3 ± 0.5 (2.5–5.9)
Serum chloride (mEq/L)	106.0 ± 3.8 (90–117)
Serum-corrected calcium (mg/L)	9.3 ± 0.6 (7–11)
Serum phosphorus (mg/dL)	3.5 ± 0.9 (1.6–10.7)
Serum triglyceride (mg/dL)	163.0 ± 92.3 (33–710)
Serum HDL (mg/dL)	51.9 ± 16.0 (19–110)
Serum LDL (mg/dL)	121.8 ± 40.8 (34–299)
Serum C-reactive protein (mg/dL)	0.5 ± 1.5 (0.1–15.7)
Proteinuria (g/day)	1.73 ± 2.32 (0.05–13.65)
Serum IgG (mg/dL)	1156.9 ± 389.8 (322–3223)
Serum IgA (mg/dL)	295.1 ± 119.8 (96–1015)
Leucocyte (10^3^/mL^3^)	6526 ± 2199 (1900–22,800)
Hemoglobin (g/dL)	12.2 ± 2.3 (7–17.2)
Platelet (10^4^/µL)	232.5 ± 73.5 (19.4–735)

Median (min–max) and mean ± standard deviation are shown

*BMI* body mass index, *eGFR* estimated glomerular filtration rate, *HDL* high-density lipoprotein, *LDL* low-density lipoprotein

## DC abs. and quantile analysis

The mean abs. of tDCs, mDCs, and pDCs were 1421.4 ± 366.0 (326–2766) cells/10^2^ µL, 702.1 ± 336.9 (61–1952) cells/10^2^ µL, and 415.5 ± 219.7 (0–1192) cells/10^2^ µL, respectively. The relative numbers of tDCs, mDCs, and pDCs (% of leukocytes) were 0.24 ± 0.09 (0.04–0.66)%, 0.12 ± 0.07 (0.01–0.51)%, and 0.07 ± 0.04 (0–0.28)%.

Quantile analysis showed that the level of daily proteinuria increased with decreasing blood mDC abs., with mean proteinuria levels (g/day) of 2.45, 1.68, 1.68, 1.10 for the mDC abs. quartiles of ≤ 445, < 686, < 907, and ≥ 907 cells/10^2^ µL (*p* = 0.0277), respectively (Table 3a). Compared to patients with the highest mDC abs., patients with the lowest mDC abs. were more often female. The lowest mDC abs. was also associated with lower serum albumin, higher C-reactive protein, a higher level of daily proteinuria and lower hemoglobin (Table 3a).

**Table 3:** Quartile analysis by each abs. of mDCs (Table 3a), pDCs (Table 3b) and tDCs (Table 3c)

(a)	mDC quartiles (cells/10^2^ µL)												
	≤ 445 (*n* = 53)	445–686 (*n* = 52)			686–907 (*n* = 53)			≥ 907 (*n* = 52)			*p* for trend		
Age (years)	58.7 ± 15.6	56.4 ± 18.0			60.7 ± 14.8			54.5 ± 16.2			0.2196		
Male, *n* (%)	23, 43.4%	28, 53.9%			29, 54.7%			41, 78.9%			0.0005		
Diabetes mellitus nephropathy, *n* (%)	9, 17.0%	5, 9.6%			6, 23.1%			6, 11.5%			0.4674		
BMI (kg/m^2^)	23.1 ± 4.1	23.0 ± 3.8			24.1 ± 3.9			23.8 ± 3.3			0.3598		
Glucocorticoids user, *n* (%)	6, 11.3%	8, 15.4%			7, 13.2%			3, 5.8%			0.3432		
Serum albumin (g/dL)	3.3 ± 0.8	3.6 ± 0.8			3.6 ± 0.7			3.7 ± 0.6			0.0162		
Serum urea nitrogen (mg/dL)	29.5 ± 21.7	23.0 ± 16.1			24.8 ± 15.6			22.0 ± 14.8			0.1231		
Serum creatinine (mg/dL)	2.0 ± 1.8	1.7 ± 1.3			1.8 ± 1.6			1.5 ± 0.7			0.3045		
eGFR (mL/min/1.73 m^2^)	43.5 ± 30.9	51.3 ± 30.9			44.3 ± 25.0			49.4 ± 24.6			0.4068		
Serum sodium (mEq/L)	141.0 ± 2.7	141.1 ± 2.2			141.9 ± 2.2			142.0 ± 2.6			0.0638		
Serum potassium (mEq/L)	4.21 ± 0.65	4.25 ± 0.55			4.40 ± 0.49			4.43 ± 0.47			0.0899		
Serum chloride (mEq/L)	105.0 ± 4.1	106.4 ± 3.5			106.1 ± 3.2			106.5 ± 4.3			0.173		
Serum-corrected calcium (mg/L)	9.3 ± 0.5	9.3 ± 0.7			9.4 ± 0.6			9.4 ± 0.5			0.6508		
Serum phosphorus (mg/dL)	3.7 ± 1.2	3.5 ± 0.8			3.6 ± 0.6			3.4 ± 0.8			0.1506		
Serum triglyceride (mg/dL)	146.5 ± 75.3	162.4 ± 92.8			191.0 ± 112.3			151.9 ± 80.3			0.0612		
Serum HDL (mg/dL)	52.8 ± 18.5	53.9 ± 14.7			51.0 ± 17.9			50.1 ± 12.4			0.6201		
Serum LDL (mg/dL)	126.7 ± 46.2	120.0 ± 38.4			120.1 ± 36.4			120.5 ± 42.3			0.8017		
Serum C-reactive protein (mg/dL)	1.1 ± 2.6	0.4 ± 1.0			0.3 ± 0.4			0.3 ± 0.5			0.0116		
Proteinuria (g/day)	2.5 ± 3.2	1.7 ± 2.1			1.7 ± 2.1			1.1 ± 1.2			0.0277		
Serum IgG (mg/dL)	1240.0 ± 515.0	1114.2 ± 359.3			1188.4 ± 354.7			1082.7 ± 281.3			0.1531		
Serum IgA (mg/dL)	288.1 ± 147.0	290.2 ± 117.1			301.0 ± 98.6			300.9 ± 113.8			0.9162		
Leucocyte (10^3^/mL^3^)	6.59 ± 2.91	6.28 ± 1.87			6.35 ± 1.79			6.88 ± 2.05			0.5003		
Hemoglobin (g/dL)	11.5 ± 2.3	12.1 ± 2.5			12.2 ± 2.1			12.9 ± 2.2			0.0229		
Platelet (10^4^/µL)	232.9 ± 72.6	237.1 ± 96.4			217.9 ± 70.0			242.2 ± 46.5			0.3641		

(b)	pDC quartiles (cells/10^2^ µL)												
	≤ 256 (*n* = 52)		256–417 (*n* = 53)			417–560 (*n* = 52)			≥ 560 (*n* = 53)			*p* for trend	
Age (years)	62.9 ± 15.8		59.2 ± 15.6			57.4 ± 16.2			51.1 ± 15.5			0.0018	
Male, *n* (%)	26, 50.0%		30, 56.6%			30, 57.7%			35, 66.0%			0.1065	
Diabetes mellitus nephropathy, *n* (%)	6, 11.5%		9, 15.0%			6, 11.5%			5, 9.4%			0.5583	
BMI (kg/m^2^)	23.0 ± 3.7		23.4 ± 3.5			23.9 ± 4.2			23.7 ± 3.7			0.6773	
Glucocorticoids user, *n* (%)	5, 9.6%		8, 15.1%			4, 7.7%			7, 13.2%			0.8636	
Serum albumin (g/dL)	3.4 ± 0.8		3.6 ± 0.7			3.6 ± 0.7			3.6 ± 0.8			0.2038	
Serum urea nitrogen (mg/dL)	32.6 ± 20.9		25.6 ± 18.4			22.4 ± 10.2			19.0 ± 15.6			0.0004	
Serum creatinine (mg/dL)	2.3 ± 1.7		1.8 ± 1.2			1.7 ± 1.2			1.3 ± 1.0			0.0151	
eGFR (mL/min/1.73 m^2^)	35.8 ± 25.6		45.7 ± 26.6			45.3 ± 28.1			61.4 ± 26.1			< 0.0001	
Serum sodium (mEq/L)	141.2 ± 2.9		141.7 ± 2.6			141.8 ± 2.0			141.2 ± 2.3			0.4612	
Serum potassium (mEq/L)	4.3 ± 0.6		4.3 ± 0.6			4.3 ± 0.6			4.3 ± 0.5			0.9639	
Serum chloride (mEq/L)	106.3 ± 4.1		106.1 ± 4.5			106.2 ± 3.5			105.3 ± 3.0			0.6347	
Serum-corrected calcium (mg/L)	9.3 ± 0.6		9.3 ± 0.5			9.3 ± 0.6			9.4 ± 0.5			0.9704	
Serum phosphorus (mg/dL)	3.7 ± 1.3		3.6 ± 0.8			3.3 ± 0.6			3.5 ± 0.6			0.1194	
Serum triglyceride (mg/dL)	171.3 ± 85.6		147.8 ± 71.9			184.4 ± 113.9			149.1 ± 90.6			0.1189	
Serum HDL (mg/dL)	48.1 ± 14.1		51.4 ± 16.2			54.0 ± 16.0			54.2 ± 17.4			0.1734	
Serum LDL (mg/dL)	131.4 ± 48.0		115.7 ± 34.2			119.3 ± 43.6			120.9 ± 35.5			0.2319	
Serum C-reactive protein (mg/dL)	1.2 ± 2.7		0.4 ± 0.6			0.2 ± 0.4			0.2 ± 0.5			0.0015	
Proteinuria (g/day)	2.4 ± 3.1		1.3 ± 1.5			1.6 ± 1.9			1.7 ± 2.4			0.1045	
Serum IgG (mg/dL)	1267.5 ± 524.4		1110.3 ± 298.8			1138.3 ± 325.7			1113.2 ± 361.7			0.125	
Serum IgA (mg/dL)	306.5 ± 120.0		302.2 ± 158.0			292.9 ± 88.8			278.8 ± 101.8			0.6494	
Leucocyte (10^3^/mL^3^)	6.88 ± 3.01		6.07 ± 1.80			6.69 ± 1.91			6.47 ± 1.84			0.2703	
Hemoglobin (g/dL)	11.3 ± 2.2		11.9 ± 2.6			12.2 ± 2.2			13.3 ± 1.9			0.0001	
Platelet (10^4^/µL)	235.3 ± 100.3		237.1 ± 63.3			222.1 ± 60.1			235.4 ± 64.6			0.7057	

(c)	tDC quartiles (cells/10^2^ µL)												
	≤ 1216 (*n* = 52)			1216–1439 (*n* = 53)			1439–1637 (*n* = 52)			≥ 1637 (*n* = 53)			*p* for trend
Age (years)	62.5 ± 15.1			60.7 ± 16.6			55.1 ± 14.6			52.2 ± 16.8			0.0031
Male, *n* (%)	20, 38.5%			26, 49.1%			34, 65.4%			41, 77.4%			< 0.0001
Diabetes mellitus nephropathy, *n* (%)	8, 15.4%			7, 13.2%			7, 13.5%			4, 7.6%			0.2511
BMI (kg/m^2^)	22.8 ± 4.2			23.5 ± 4.0			23.6 ± 3.3			23.9 ± 3.6			0.5328
Glucocorticoids user, *n* (%)	7, 13.5%			8, 15.1%			5, 9.6%			4, 7.6%			0.2356
Serum albumin (g/dL)	3.3 ± 0.7			3.6 ± 0.6			3.7 ± 0.8			3.6 ± 0.7			0.0584
Serum urea nitrogen (mg/dL)	31.6 ± 19.7			24.7 ± 17.0			20.1 ± 14.0			23.1 ± 16.7			0.0061
Serum creatinine (mg/dL)	2.1 ± 1.7			1.8 ± 1.4			1.4 ± 1.2			1.6 ± 1.2			0.0989
eGFR (mL/min/1.73 m^2^)	37.7 ± 28.6			46.4 ± 29.3			54.9 ± 25.7			49.5 ± 26.1			0.0152
Serum sodium (mEq/L)	141.4 ± 3.0			141.0 ± 1.8			142.3 ± 2.0			141.3 ± 2.6			0.0341
Serum potassium (mEq/L)	4.3 ± 0.6			4.3 ± 0.6			4.3 ± 0.5			4.4 ± 0.5			0.4635
Serum chloride (mEq/L)	105.8 ± 3.9			106.2 ± 3.7			106.9 ± 3.7			105.0 ± 3.8			0.0681
Serum-corrected calcium (mg/L)	9.3 ± 0.5			9.2 ± 0.5			9.5 ± 0.6			9.3 ± 0.6			0.1358
Serum phosphorus (mg/dlL)	3.8 ± 1.2			3.6 ± 0.8			3.4 ± 0.6			3.4 ± 0.7			0.063
Serum triglyceride (mg/dL)	162.0 ± 90.5			147.2 ± 69.0			174.5 ± 108.7			168.5 ± 97.2			0.4692
Serum HDL (mg/dL)	47.6 ± 14.0			57.5 ± 18.3			52.8 ± 15.6			49.8 ± 14.6			0.0102
Serum LDL (mg/dL)	120.0 ± 37.9			121.3 ± 36.5			123.0 ± 48.2			123.0 ± 40.8			0.9777
Serum C-reactive protein (mg/dL)	1.1 ± 2.7			0.4 ± 0.7			0.2 ± 0.3			0.3 ± 0.5			0.0048
Proteinuria (g/day)	2.24 ± 2.58			1.36 ± 1.95			1.77 ± 2.86			1.54 ± 1.64			0.2351
Serum IgG (mg/dL)	1252.5 ± 525.5			1166.7 ± 350.6			1148.0 ± 352.8			1061.9 ± 276.3			0.0956
Serum IgA (mg/dL)	302.3 ± 157.0			298.0 ± 111.1			274.6 ± 83.7			305.1 ± 116.8			0.5536
Leucocyte (10^3^/mL^3^)	6.59 ± 2.85			6.08 ± 1.85			6.79 ± 1.88			6.65 ± 2.07			0.3718
Hemoglobin (g/dL)	11.1 ± 2.2			11.9 ± 2.1			12.9 ± 2.1			12.9 ± 2.5			< 0.0001
Platelet (10^4^/µL)	234.4 ± 98.1			225.6 ± 67.8			233.6 ± 59.3			236.4 ± 64.7			0.8839

Quartile analysis showed that the level of daily proteinuria increased with decreasing blood mDC abs., with mean proteinuria levels (g/day) of 2.45, 1.68, 1.68, 1.10 for the mDC abs. quartiles of ≤ 445, < 686, < 907, ≥ 907 cells/10^2^ µL (*p* = 0.0277), respectively. Compared to patients with the highest mDC abs., patients with the lowest mDC abs. were more often female. The lowest mDC abs. was also associated with lower serum albumin, higher C-reactive protein, a higher level of daily proteinuria and lower hemoglobin **(**Table 3a**)**. Compared to patients with the highest pDC abs., patients with the lowest pDC abs. were older and had lower eGFR, higher BUN, higher serum Cr, higher C-reactive protein and lower hemoglobin **(**Table 3b**)**. Compared to patients with the highest tDC abs., patients with the lowest tDC abs. were older and more often female and had lower eGFR, higher BUN, higher C-reactive protein and lower hemoglobin **(**Table 3c**)**

Compared to patients with the highest pDC abs., patients with the lowest pDC abs. were older and had lower eGFR, higher BUN, higher serum Cr, higher C-reactive protein and lower hemoglobin (Table 3b).

Compared to patients with the highest tDC abs., patients with the lowest tDC abs. were older and more often female and had lower eGFR, higher blood urea nitrogen (BUN), higher C-reactive protein and lower hemoglobin (Table 3c).

### Associations between clinical factors and DC abs.

As shown in Table 4, univariate and multivariate-adjusted regression analyses were used to assess the independent associations between DC subsets (tDCs, mDCs, and pDCs) and explanatory variables.

**Table 4:** Associations between clinical factors and DC abs.

	tDC				mDC				pDC			
	Univariable model		Multivariable model		Univariable model		Multivariable model		Univariable model		Multivariable model	
—	95% CI	*p* value	95% CI	*p* value	95% CI	*p* value	95% CI	*p* value	95% CI	*p* value	95% CI	*p* value
Age (years)	− 7.0 to − 0.9	0.0105	− 5.29 to 1.49	0.2696	− 3.74 to 1.92	0.5277	− 3.16 to 3.12	0.9901	− 5.3 to − 1.7	0.0001	− 3.3 to 0.7	0.1934
Male gender	75.8—271.1	0.0006	54.5—253.6	0.0026	66.2–250.5	0.0008	66.2–250.5	0.0008	− 20.5 to 100.3	0.1942	− 41.9 to 75.4	0.5748
Alb (g/dL)	− 18.5 to 120.0	0.1498	− 160.0 to 22.7	0.1398	26.7–152.4	0.0055	− 86.2 to 82.9	0.9689	10.2–92.6	0.0147	− 66.5 to 41.1	0.6423
eGFR (mL/min/1.73 m^2^)	0.86–4.36	0.0037	− 1.28 to 3.02	0.4269	− 1.25 to 2.04	0.6355	− 3.19 to 0.79	0.2354	1.54–3.57	< 0.0001	0.14–2.67	0.0298
Proteinuria (g/day)	− 42.6 to 0.2	0.0521	− 48.7 to 3.7	0.0925	− 53.1 to − 14.4	0.0007	− 57.0 to − 8.5	0.0084	− 25.3 to 0.4	0.0576	− 19.3 to 11.6	0.6223
C-reactive protein (mg/dL)	− 88.8 to − 21.8	0.0013	− 80.5 to − 13.4	0.0063	− 67.0 to − 4.6	0.0249	− 60.4 to 1.7	0.0638	− 59.9 to − 20.1	0.0001	− 49.4 to − 9.9	0.0034
Hemoglobin (g/dlL)	18.4–59.8	0.0003	− 11.3 to 43.5	0.2489	6.8–45.6	0.0083	− 14.0 to 36.7	0.3772	17.7 to 42.1	< 0.0001	− 3.3 to 29.0	0.1184

Univariate and multivariate-adjusted regression analyses were used to assess the independent associations between DC subsets (tDCs, mDCs, and pDCs) and explanatory variables. The primary variables were tDC abs., mDC abs., and pDC abs., and the explanatory variables were determined according to their univariate relationship or importance within the explanatory variables as follows: age, gender, albumin, eGFR, level of daily proteinuria, C-reactive protein, and hemoglobin. Multivariate-adjusted regression analysis for mDC abs. revealed an independent negative association with the level of proteinuria (*t* = − 2.66, 95% CI − 57.0 to − 8.5, *p* = 0.0084) and a positive association with male gender (*t* = 3.39, 95% CI 66.2–250.5, *p* = 0.0008) after adjusting for age, serum albumin, eGFR, C-reactive protein, and hemoglobin. Multivariate-adjusted regression analysis for pDC abs. revealed an independent positive association with eGFR (*t* = 2.19, 95% CI 0.14–2.67, *p* = 0.0298) and a negative association with C-reactive protein (*t* = − 2.96, 95% CI − 49.4 to − 9.9, *p* = 0.0034) after adjusting for age, male gender, serum albumin, level of proteinuria, and hemoglobin. Multivariate-adjusted regression analysis (least-squares method) for tDC abs. revealed an independent positive association with male gender (*t* = 3.05, 95% CI 54.5–253.6, *p* = 0.0026) and a negative association with C-reactive protein (*t* = − 2.76, 95% CI − 80.5 to − 13.4, *p* = 0.0063) after adjusting for age, serum albumin, eGFR, level of proteinuria, C-reactive protein, and hemoglobin

The primary variables were tDCs abs., mDCs abs. and pDCs abs., and the explanatory variables were determined according to their univariate relationship or importance within the explanatory variables as follows: age, gender, albumin, eGFR, level of daily proteinuria, C-reactive protein and hemoglobin.

Multivariate-adjusted regression analysis for mDCs abs. revealed an independent negative association with the level of proteinuria (*t* = − 2.66, 95% CI − 57.0 to − 8.5, *p* = 0.0084) and a positive association with male gender (*t* = 3.39, 95% CI 66.2–250.5, *p* = 0.0008) after adjusting for age, serum albumin, eGFR, C-reactive protein, and hemoglobin. Although serum albumin, C-reactive protein and hemoglobin were factors associated with mDCs abs. in univariate logistic regression analyses, these factors were not independent risk factors in multivariate-adjusted regression analysis.

Multivariate-adjusted regression analysis for pDCs abs. revealed an independent positive association with eGFR (*t* = 2.19, 95% CI 0.14–2.67, *p* = 0.0298) and a negative association with C-reactive protein (*t* = − 2.96, 95% CI − 49.4 to − 9.9, *p* = 0.0034) after adjusting for age, male gender, serum albumin, level of proteinuria, and hemoglobin. Although age and hemoglobin were factors associated with pDCs abs. in univariate analyses, these factors were not independent risk factors in multivariate-adjusted logistic regression analysis.

Again, multivariate-adjusted regression analysis (least-squares method) for tDCs abs. revealed an independent positive association with male gender (*t* = 3.05, 95% CI 54.5–253.6, *p* = 0.0026) and a negative association with C-reactive protein (*t* = − 2.76, 95% CI − 80.5 to − 13.4, *p* = 0.0063) after adjusting for age, serum albumin, eGFR, level of proteinuria, C-reactive protein, and hemoglobin.

Although younger age, higher level of eGFR, hemoglobin and lower level of phosphate were factors associated with the tDCs abs. in univariable logistic regression analyses, these factors were not independent risk factors in multivariate-adjusted regression analysis.

Diabetic nephropathy, BMI, glucocorticoid use, sodium, chloride, corrected calcium, phosphate, triglyceride, HDL, LDL, IgG, IgA, leukocytes and platelets were not associated with tDCs, mDCs or pDCs.

## Discussion

The present study demonstrated four factors (proteinuria, eGFR, C-reactive protein, and gender) independently associated with circulating mDC abs., pDC abs. or tDC abs. by multivariate regression analysis as follows. First, we revealed that the circulating mDC abs. was negatively associated with the level of proteinuria, independent of eGFR. These data suggest that mDCs may recruit from circulating blood to injured kidneys exhibiting heavy proteinuria, independent of the CKD stage. For accuracy, the level of proteinuria was thoroughly measured by 24-h urine collection from patients in their admitted condition, not by spot-measuring g/Cr. This accurate measurement may contribute to the result. Second, the circulation of pDC abs. had a positive association with eGFR and no association with proteinuria. This suggests that pDCs are recruited into the kidney as kidney function declines, independent of the level of proteinuria. Thus, CKD stage and heavy proteinuria may regulate the distribution of each DC subset separately. Third, C-reactive protein was negatively associated with both pDC abs. and tDC abs. Fourth, male gender was positively associated with both mDC abs. and tDC abs.

A possible mechanism of the decrease in circulating DCs involves either DC death, a distribution shift from circulating blood to organs, or the other unknown mechanism in some kidney disease without inflammatory cell infiltration. DCs may die following infection, as in malaria [18] or severe sepsis [19], where infected DCs have been shown to undergo apoptosis. However, this mechanism seems unlikely to occur in the present CKD patients who did not have any complications of infection. Concerning the distribution shift, immature circulating mDCs are recruited to the site where the immunological response should occur, such as inflammation, tumor, hypoxic lesions, and atherosclerotic lesions, and differentiate into mature DCs [20]. For example, Yilmaz et al. [6] reported a significant reduction in circulating mDCs without a reduction in pDCs in patients with coronary artery disease. The emergence of mDC precursors in vulnerable plaques suggests their recruitment into atheromata as a possible reason for their decrease in blood. Several reports have suggested that circulating mDCs are stimulated to migrate into the kidney glomeruli and interstitial region, which may lead to a decrease in circulating mDC abs [11]. In fact, both mDCs and pDCs were identified within the tubulointerstitium in the renal biopsies, and increased tubulointerstitial recruitment of human DCs has also been reported in lupus nephritis [3, 4], acute tubulointerstitial nephritis (ATIN) [21], renal fibrosis and CKD [22, 23]. Fiore et al. reported that mDCs and pDCs that were decreased at the circulating level were recruited within the tubulointerstitial lesion in patients with active lupus nephritis. Segerer et al. [23] reported that mDC marker (DC-SIGN)-positive cells correlated significantly with serum Cr but not with proteinuria by simple univariate analysis in 55 renal biopsies. Their sample size might be small to detect the differences between DC-SIGN-positive cells and proteinuria, and they did not measure circulating DC subsets in the blood, so the association between circulating mDCs and proteinuria or DC-SIGN-positive cells in the kidney was not analyzed. The recruitment of DCs to the kidney with heavy proteinuria may be controlled by the sequential action of different chemokines as follows: the expression of monocyte chemotactic protein (MCP) by cells lining blood vessels, the production of MIP-3alpha/CCL20 by epithelial cells and the upregulation of CCR6 in response to the tissue environment [24]. Thus, chemoattractant-stimulated mDCs may migrate from circulating blood to the tubulointerstitial area in patients with heavy proteinuria. Our results suggest that this recruitment may be a common phenomenon in various kidney diseases with heavy proteinuria.

In the present study, all DC subsets showed a negative association with C-reactive protein, though a weak association was observed between C-reactive protein and mDCs (95% CI − 60.4 to 1.7, *p* = 0.0638). Similar to our study, several reports have shown a decrease in the mDC or/and pDC count in inflammatory diseases, such as septic shock [25], influenza A [26], and C-reactive protein-producing carcinoma [27].

The present multivariate regression analysis (210 patients) supports the proposal that renal dysfunction (decrease in eGFR) could have a negative effect on at least pDCs but not mDCs in patients with predialysis CKD, such as that in a report by Hesselink et al. [12]. Several reports have shown a reduction in the number of circulating mDCs in patients with CKD both receiving RRT and not receiving RRT [12–14]. Paul et al. [14] reported higher hsCRP concentration and the presence of diabetes mellitus had a significant negative influence on DC count but not age and eGFR. We speculate the reason were some differences of patient characteristics between ours and theirs. They selected only the patients with smaller range of eGFR (30–60 ml/min/1.73 m^2^) than this study (4.5–133.2 ml/min/1.73 m^2^), so that they could not detect a significant differences between DC number and eGFR. And this study involved only the patients who received kidney biopsy and the frequency of diabetic nephropathy was small (39.7% in Pauls’ vs 12.4% in this study) so that this study might not be able to detect a significant differences between DC number and the presence of diabetes mellitus. These discrepancy of patient characteristics might influence the results.

Human blood mDCs and pDCs express estrogen receptors (ERs) and respond to estrogens, suggesting that estrogens may contribute to the sex differences in immunity by regulating DC biology [28]. Among our 210 patients, male gender was positively associated with both mDC abs. and tDC abs. but was not associated with pDC abs. by multivariate regression analysis. In a healthy population, Orsini G et al. showed a similar male-dominant increase in mDC and tDC numbers, but not in pDC number, by univariate analysis in a relatively smaller sample number (44 males and 52 females), though they did not confirm their findings by a multivariate regression model [29].

Orsini et al. also observed that only the pDC abs. significantly decreased with increasing age by univariate analysis, whereas the mDC abs. did not show any age correlation in the subjects over 20 years [29]. We also showed that aging was associated with a decline in pDC number but not in mDC number by univariate analysis as reported by Jing et al. [30]. However, multivariate regression analysis showed no significant differences between age and each DC subset in our study.

### Limitation

We conducted a cross-sectional study of 210 patients with various kidney disease and we could not detect disease specific relationship between proteinuria and circulating DCs in each renal disease.

This study included a relatively smaller number of patients with diabetic nephropathy compared to the usual CKD population given their relatively lower indication for kidney biopsy, which may influence the results. Thus, further large-scale study will be needed to clarify the relationship in each renal disease.

This study did not involve intact healthy control subjects; however, the range of eGFR was wide (4.5–133.2 ml/min/1.73 m^2^), 16 patients with a normal eGFR > 90 ml/min/1.73 m^2^, and 23 patients with a normal daily proteinuria < 0.15 g, these patient characteristics may influence the results.

We did not detect the local mDC by immunostaining nor by histopathological findings of tubulointerstitial inflammation and we did not measure any serum concentrations of IFN, inflammatory cytokines and chemokines, so we could not clarify the mechanism by which proteinuria influences the decrease in circulating mDCs.

The measurement of both daily proteinuria and DC count was performed in 1 day simultaneously, although it would be preferable if more than two times simultaneous measurement of both was possible.

## Conclusion

We first reported that circulating mDC abs. had a negative association with the level of proteinuria and no association with eGFR in CKD patients not receiving dialysis. C-reactive protein was negatively associated with both the pDC abs. and tDC abs., and male gender was positively associated with both the mDC abs. and tDC abs. in these patients.

Notably, we observed a strong reduction in mDCs, while the pDCs appears mostly unchanged in patients with heavy proteinuria. This is in line with several diseases where the involvement of DCs as a factor associated with the diseases was highlighted.

A decrease in the circulating mDC abs. may reflect the degree of kidney damage in CKD patients, and measurement of the blood mDC abs. may be a biomarker for renal disease activity.

## Abbreviations

DC: Dendritic cell
tDC: Total DC
mDC: Myeloid DC
pDC: Plasmacytoid DC
FACS: Fluorescence-activated cell sorting
abs.: Absolute number
CI: Confident interval
DCs: Dendritic cells
CNS: Central nervous system
CKD: Chronic kidney disease
RRT: Renal replacement therapy
eGFR: Estimated glomerular filtration rate
BMI: Body mass index
Cr: Creatinine
HDL: High-density lipoprotein
LDL: Low-density lipoprotein
NK cells: Natural killer cells
BUN: Blood urea nitrogen
HLA: Human leukocyte antigen
CD: Cluster of differentiation
IgG: Immunoglobulin G
IgA: Immunoglobulin A
IgAN: IgA nephropathy
MN: Membranous nephropathy
Minor: Minor glomerular abnormalities
CreGN: Crescentic glomerulonephritis
FSGS: Focal segmental glomerulosclerosis
MCD: Minimal change disease
ATIN: Acute tubulointerstitial nephritis
MIP: Macrophage inflammatory protein
CCL: CC chemokine ligand
CCR: CC chemokine receptor
